# The role of GPI-anchored membrane-bound alkaline phosphatase in the mode of action of Bt Cry1A toxins in the diamondback moth

**DOI:** 10.1016/j.fmre.2024.05.007

**Published:** 2024-05-27

**Authors:** Dan Sun, Qiuchen Xu, Le Guo, Yang Bai, Xuping Shentu, Xiaoping Yu, Neil Crickmore, Xuguo Zhou, Alejandra Bravo, Mario Soberón, Youjun Zhang, Zhaojiang Guo

**Affiliations:** a Zhejiang Provincial Key Laboratory of Biometrology and Inspection & Quarantine, College of Life Science, China Jiliang University, Hangzhou 310018, China; b State Key Laboratory of Vegetable Biobreeding, Department of Plant Protection, Institute of Vegetables and Flowers, Chinese Academy of Agricultural Sciences, Beijing 100081, China; c School of Life Sciences, University of Sussex, Brighton BN1 9QG, UK; d Department of Entomology, School of Integrative Biology, College of Liberal Arts & Sciences, University of Illinois Urbana-Champaign, IL 61801-3795, USA; e Departamento de Microbiología Molecular, Instituto de Biotecnología, Universidad Nacional Autónoma de México, Apdo. Postal 510-3, Cuernavaca 62250, Mexico

**Keywords:** *Bacillus thuringiensis*, *Plutella xylostella*, Membrane-bound alkaline phosphatase, ABC transporter, CRISPR/Cas9, Cry1Ac resistance

## Abstract

The insecticidal Cry proteins produced by the bacterium *Bacillus thuringiensis* (Bt) are extensively used for pest control in formulated sprays and in genetically modified crops, but resistance to Bt toxins threatens their sustainable use in agriculture. Understanding the molecular mechanisms involved in Bt pathogenesis is crucial for the development of effective resistance management strategies. Previously, we showed a strong correlation between Cry1Ac resistance in *Plutella xylostella* (L.) and down-regulation of the glycosylphosphatidylinositol (GPI)-anchored membrane-bound alkaline phosphatase (mALP) and aminopeptidase (APN) and members of the ATP-binding cassette (ABC) transporter subfamily C (ABCC), but we do not yet have a clear understanding of the relative contribution of each midgut receptor type. Here, a *P. xylostella* strain homozygous for the *PxmALP* gene knockout was generated using CRISPR/Cas9 and the results showed that this strain had a 294-fold resistance to Cry1Ac toxin and 394-fold cross-resistance to Cry1Ab. Moreover, a triple knockout strain lacking *PxmALP, PxABCC2*, and *PxABCC3* exhibited 9,660-fold resistance to Cry1Ac and 5,662-fold cross-resistance to Cry1Ab. These resistance levels surpassed those observed in the previously described double *PxABCC2* and *PxABCC3* knockout mutant, revealing a functional redundancy between ABC transporters and PxmALP*.* In addition, the activity of Cry1A toxins against Sf9 cells expressing PxmALP, PxABCC2 or PxABCC3 confirmed that each of these can act as a functional receptor. Our findings are crucial for unraveling the relative role of multiple receptors and the molecular mechanisms underlying Bt resistance in insects.

## Graphical abstract

**Figure:**
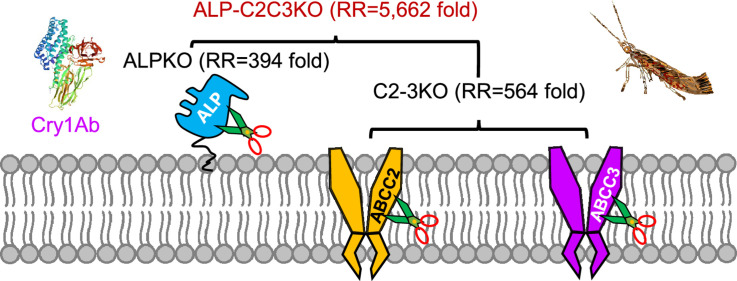

## 1 Introduction

*Bacillus thuringiensis* (Bt) is an entomopathogenic bacterium that produces different types of insecticidal proteins that show toxicity to a wide range of insect pests. The efficiency and biosafety of Bt have led to significant socio-economic benefits globally [1,2]. In 2019, the 24^th^ year of commercialization of biotech crops, 29 countries cultivated 190.4 million hectares of biotech crops [3]. Nonetheless, the long-term effectiveness of Bt products is always under threat from the evolution of insect resistance to these toxins [4,5]. So far, field-evolved practical resistance to Bt products has been reported in at least 13 insect species, comprising 11 lepidopterans and 2 coleopterans [[5], [6], [7]]. It is crucial to understand the molecular mechanisms behind Bt resistance to formulate pest management tactics that can effectively delay the evolution of resistance in the field [8].

The Cry toxin family comprises parasporal crystal proteins produced by Bt which share a three-domain structure [9]. To exert toxicity, Cry toxins must be activated by larval gut proteases and bind to specific midgut receptors on the cell membrane, where they form lytic pores that lead to insect death [8,10,11]. Various midgut membrane proteins, such as cadherin, aminopeptidase-N (APN), alkaline phosphatase (ALP), and ABC transporters, have been identified as functional receptors for Bt Cry toxins in different insect species [8,[12], [13], [14]]. In one model of the mode of action of Bt Cry1A toxins, toxin oligomerization is triggered by the interaction with a cadherin receptor. This interaction is followed by the binding of the oligomer to secondary receptors like the membrane-bound ALP (mALP), APN or an ABC transporters, facilitating membrane insertion of the oligomeric structure [15]. Recent studies have suggested that Cry proteins can display a dual mode of action whereby Bt Cry protoxins and activated toxins interact differentially with multiple receptors [[16], [17], [18]]. It is proposed that the activated Cry1Ac toxin requires interaction with cadherin and ABC transporters to induce toxicity, while the Cry1Ac protoxin interacts with GPI-anchored proteins and ABC transporters [19]. It has also been shown that the C-terminal region of Cry1A protoxins has additional binding sites for GPI-anchored APN and ALP proteins [20]. Understanding the toxicological mechanisms of Bt toxins and their interactions with insect midgut receptors is crucial for maintaining the efficacy of Bt-based pest management strategies and for ensuring their sustainable use in agriculture [21].

The diamondback moth, *Plutella xylostella* (L), is a globally devastating pest that primarily destroys cruciferous vegetables and oilseed crops [22] and was the first insect to develop resistance to Bt sprays in field conditions [23]. Previously, we showed that resistance to Cry1Ac in *P. xylostella* was not linked to the cadherin gene, but was associated with reduced expression of GPI-anchored proteins (APN and ALP) and ABC transporters (ABCC2 and ABCC3) [[24], [25], [26], [27], [28], [29], [30]]. Reports have indicated that functional redundancy between the paralogous ABCC2 and ABCC3 proteins requires both to be inactivated in order to confer significant resistance to Cry1Ac [25,31], and we have further shown redundancy between ABCC2, ABCC3, APN1 and APN3a in *P. xylostella* [32]. In this study, we wanted to extend these findings and investigate the contribution of PxmALP to the resistance phenotype. CRISPR/Cas9-based single (*PxmALP*) and triple (*PxmALP, PxABCC2* and *PxABCC3*) knockouts were constructed. Our results show that each of the three proteins could function as independent receptors and that mutating all three leads to high resistance levels against two Bt Cry1A toxins. These data show the role of multiple receptors in the mechanism of action of Bt Cry toxins.

## 2 Materials and methods

### 2.1 Insect strains and cell lines

Three *P. xylostella* strains - the susceptible DBM1Ac-S, the Bt-resistant NIL-R and the knockout strain C2-3KO were utilized in this study [33,34]. C2-3KO strain was derived from DBM1Ac-S by the simultaneous deletion of *PxABCC2* and *PxABCC3* genes, leading to a 4,482-fold resistance level to Cry1Ac protoxin compared to the susceptible DBM1Ac-S strain [32]. All *P. xylostella* strains were mass-reared at 25 °C with 65% relative humidity and a photoperiod of 16:8 (light: dark) on Jing Feng No. 1 cabbage (*Brassica oleracea* var. *capitata*), and adults provided with a 10% honey/water solution.

The *Spodoptera frugiperda* cell line (Sf9) was cultured in Sf-900 insect cell medium supplemented with 10% fetal bovine serum (Invitrogen), 100 U penicillin and 100 µg/mL streptomycin at 28 °C. For the expression of recombinant proteins cells were grown in Grace's insect medium (Invitrogen).

### 2.2 In vitro synthesis of single guide RNA (sgRNA)

**Fig. 1:**
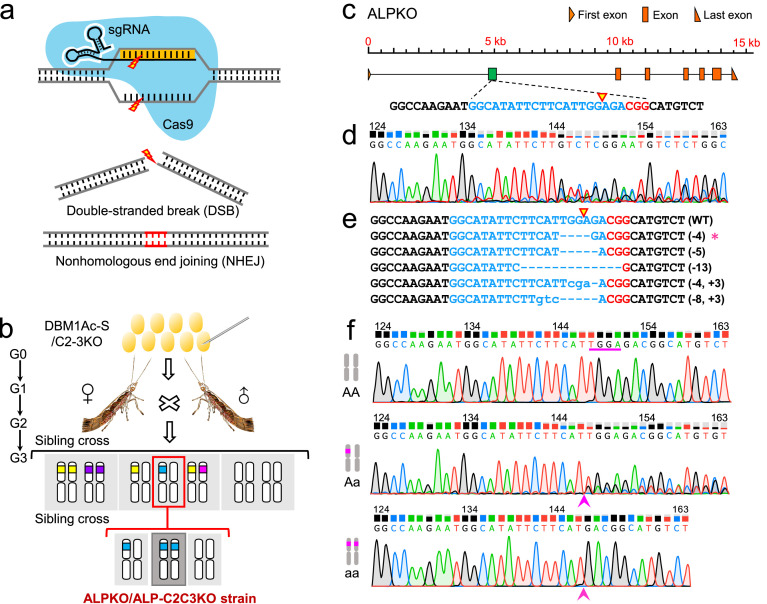
**CRISPR/Cas9-mediated genome engineering strategy in *P. xylostella*.** (a) CRISPR/Cas9-mediated genome editing and the NHEJ-based repair pathway. (b) Crossing scheme to generate homozygous single and triplex mutant strains. For different genotypes, white columns represent autosomes, and the boxes with different colors indicate the autosomal regions with *PxmALP* deleted. (c) The design of sgRNA targeting exon 2 of *the PxmALP* gene. The genomic structure is drawn to scale. The sgRNA target sequences are marked in blue and the PAM site in red; the cleavage site is showed by an open triangle. (d) CRISPR/Cas9-induced gene mutations in G0 individuals as ascertained by direct sequencing. (e) The exact mutation genotypes in G1 individuals as identified by TA cloning and sequencing. Among these different mutation genotypes, deleted bases are indicated as dashes, and inserted bases as lowercase letters. The number of deleted and inserted bases is shown at the right of each allele. The asterisk represents the selected monoallelic mutation used for crossing to yield G2 progeny. (f) Representative chromatograms of direct sequencing of the PCR products from wild type (top), heterozygous (middle), and homozygous mutants (bottom) of the *PxmALP* gene. The CRISPR/Cas9-induced 4-bp deletion (TGGA) in exon 2 of the *PxmALP* gene is labeled by the pink line and arrow.

A sgRNA was designed in exon 2 of *PxmALP* gene following the 5′-GGN_18_NGG-3′ principle using the CRISPR RGEN tool Cas-Designer (http://www.rgenome.net/cas-designer/) (Fig. 1c). The sgRNA target sequence underwent off-target evaluation using the CRISPR RGEN tool (http://www.rgenome.net/cas-offinder/) by BLAST searching the GenBank database (https://www.ncbi.nlm.nih.gov/), revealing no potential off-target sites. Double-strand DNA oligonucleotides were synthesized using template-free fusion PCR technology, containing the T7 polymerase binding site, 20-bp sgRNA target sequences, and the universal stem-loop tracrRNA sequence were designed as an upstream primer (Table S1). PCR reaction conditions and programs were as previously reported [25]. Subsequently, the purified PCR product served as a template for sgRNA synthesis by *in vitro* transcription using the MEGAscript Transcription Kit (Ambion), a further purification step was performed using the MEGAclear Kit (Ambion). The sgRNA sample was used immediately or stored at −80 °C until needed.

### 2.3 Embryo collection and microinjection

Freshly laid eggs from the DBM1Ac-S and C2-3KO strains were separately collected on dry microscope slides (24 mm × 50 mm) precoated with fresh cabbage leaf juice. A 1 nL mixture of Cas9 protein (100 ng/µL) (Thermo Fisher Scientific) and sgRNA (300 ng/µL) targeting *PxmALP* was microinjected into the individual embryos at the posterior pole within 2 h after oviposition. The instruments and injection procedures were as previously described [25], and the injected eggs were incubated at 25 °C with 65% relative humidity until hatching.

### 2.4 Mutagenesis detection

To accurately determine the genotype mutations of *PxmALP*, an efficient and non-destructive testing method combined with sequencing was employed as previously described [25]. Briefly, the genomic DNA (gDNA) fragment flanking the sgRNA target site of *PxmALP* was amplified from the exuviates of 4th-instar *P. xylostella* larvae using specific primers (ALP-F/ALP-R) (Table S1). The PCR product was then directly sequenced using ALP-F as a sequencing primer. Heterozygous mutations were indicated if the sequencing chromatogram exhibited a cluster of double peaks spanning the sgRNA target site. Additionally, the PCR products underwent TA cloning and were sequenced to confirm the precise mutation (Fig. 1d–f).

### 2.5 Creation of homozygous single and triple knockout strains

To construct the stable homozygous single and triple mutant strains, the mixture of Cas9 protein and sgRNA targeting *PxmALP* were separately injected into an individual embryo of the DBM1Ac-S and C2-3KO strains to obtain G0 progeny. Mutant moths identified by direct sequencing were sib-mated to generate G1 progeny, subsequently, the exact indel types of alleles were verified based on the results of direct sequencing and TA cloning/sequencing in G1 progeny. Heterozygous individuals with the same allelic mutation of each gene were in-crossed to produce G2 offspring. Eventually, the G2 progeny with homozygous mutant alleles were sibling-crossed to produce the stable homozygous mutant strains ALPKO and ALP-C2C3KO in G3 offspring (Fig. 1b).

### 2.6 Cry1Ac toxin preparation and bioassay

The methods for preparing Cry1A protoxins and trypsin-activated Cry1A toxins were as described previously [[35], [36], [37]]. The Cry1A toxin was maintained in 50 mM Na_2_CO_3_ (pH 9.6)/0.5% triton X-100 and stored at −20 °C until needed.

The toxicity of Bt Cry1A protoxin was assessed in 72 h bioassays performed using a leaf-dip method as described elsewhere [26]. Ten 3rd-instar larvae were tested against seven toxin concentrations and bioassays were repeated three times. Larval mortality was recorded for each strain only if the control mortality without toxin was no more than 5%. After bioassays, the LC_50_ values (the concentration that killed 50% of the tested larvae) and 95% CL (95% fiducial limits of LC_50_ values) values were calculated by Probit analysis using POLO Plus 2.0 software (LeOra Software). The LC_50_ values of pairwise comparison were considered as significantly different if their 95% CLs did not overlap.

### 2.7 Construction of recombinant plasmids

The full-length cDNA sequences of *PxABCC2-3* and *PxmALP* genes were cloned from DBM1Ac-S larvae via amplification by high-fidelity PrimeSTAR Max DNA polymerase (TaKaRa) with gene-specific primers (Table S1). Subsequently, the PCR products of these four genes were purified with the DNA Clean-up Kit (CWBIO), finally subcloned into the pUC-57 cloning vector and sequenced. The verified positive clones were digested with *Hind*III and *Nhe*I and then these DNA fragments were inserted into the pie2-EGFP-N1 expression vector to generate three recombinant plasmids (pie2-EGFP-*PxABCC2*, pie2-EGFP-*PxABCC3*, and pie2-EGFP-*PxmALP*). All the recombinant plasmids contained the pie2 promotor and a fusion to EGFP. The negative control plasmid only expressed EGFP.

### 2.8 Heterologous expression and ligand binding assay

We had previously confirmed that Cry1Ac can bind to the receptor paralogs *PxABCC2* and *PxABCC3* and that the susceptibility to Cry1Ac was affected when these proteins were ectopically expressed in *Spodoptera frugiperda* (Sf9) cells [38]. In this study, the recombinant PxmALP protein was also transiently expressed in Sf9 cells. Initially, Sf9 cells were cultured in sterile six-well plates (Costar) at a density of 1 × 10^6^ cells/well in Sf-900 serum-free medium (Gibco) with 10% fetal bovine serum (FBS) (Invitrogen). Plasmid transfection into Sf9 cells was carried out using Cellfectin II Reagent (Invitrogen) at 2 µg/well. The specific interaction between PxmALP recombinant protein and Cry1A toxins was determined by immunolocalization 36 h post-transfection. The Sf9 cells expressing the PxmALP recombinant protein were incubated with the trypsin-activated Cry1A toxin (100 mg/L) at 27 C for 2 h. Following incubation, the cells were washed three times with PBS buffer (pH 7.4) (3 min each time), fixed with 4% paraformaldehyde for 15 min, permeabilized with 1% Triton X-100 for 20 min, and blocked with 5% BSA at 25 °C for 1 h. Subsequently, the cells were sequentially incubated with primary rabbit polyclonal anti-Cry1A antibody (1:100 dilution) and then with the goat anti-rabbit secondary antibody conjugated with Alexa Fluor 555 (1:500 dilution, Abcam) for 1 h at 27 °C. Finally, the unconjugated antibodies were removed by washing with PBS buffer as described above and the cells placed on microscope slides, mounted with coverslips, and observed under an LSM 700 confocal laser scanning inverted fluorescence microscope (Carl Zeiss).

### 2.9 Cytotoxicity detection

Cytotoxicity assays were performed by incubating Sf9 cells expressing PxABCC2-3/PxmALP recombinant proteins with Cry1A toxins (100 µl, 0.1 nM-1 mM) for 24 h at 27 °C. Subsequently, the culture medium was replaced with a new 100 µl medium containing 10 µl WST-8 from the Cell Counting Kit-8 (CCK-8) (Dojindo). CCK-8 assays were performed at 27 °C for 2 h, and the absorbance was measured at 450 nm. The control group contained 100 µl culture medium and 10 µl CCK-8 without cells, while the treatment group included 100 µl of culture medium with Cry1A-treated Sf9 cells and 10 µl CCK-8. The relative viability of cells was measured in relation to the untreated Sf9 cells, which was defined as 100%. The experiments were repeated six times and data were statistically analyzed by using a one-way ANOVA with Holm-Sidak’s tests (overall significance level = 0.05).

### 2.10 Inheritance and genetic complementation analysis

To determine the inheritance of Bt Cry1Ac resistance in both KO strains, we measured the response to Cry1Ac following a crossing strategy between two pairs of the DBM1Ac-S, C2-3KO, ALPKO, and ALP-C2C3KO strains. The larval mortality of these four parental strains (50 larvae from each strain) and their F1 offspring (100 larvae from their progeny) were screened with a diagnostic dose (10 mg/L) of Cry1Ac protoxin, which would kill all heterozygous F1 larvae. Subsequently, the dominance (*h*) of Cry1Ac resistance was calculated as follows: *h* = (C-S)/(R-S), where C, S, R represent the larval survival rate of the F1 hybrid progeny, the susceptible DBM1Ac-S, and the KO-strains. The value of *h* changes from 0 (completely recessive resistance) to 1 (completely dominant resistance) [39].

Subsequently, bioassays were conducted on hybrid progeny from the various strains to assess genetic-based variation in resistance to Cry1Ac toxin. Offspring exhibit resistance to Bt Cry1Ac toxin when recessive resistance alleles are located in the same locus, while susceptibility to Bt Cry1Ac toxin is observed in progeny when recessive resistance alleles are located in different loci.

### 2.11 Fitness costs analysis

The potential fitness costs of the single and triple KO mutant strains were analyzed by comparing the biological parameters of larval duration time, pupation rate, pupal weight, pupation duration time, eclosion percentage, and adult longevity. Larvae from susceptible DBM1Ac-S were used as a control. Each test was replicated five times with ten larvae per replicate. Any statistically significant differences in these biological parameters between control and genetic editing strains were evaluated by one-way ANOVA with Holm-Sidak’s test (overall significance level = 0.05).

## 3 Results

### 3.1 Construction of homozygous single and triple KO strains

Previously we demonstrated that high-level resistance to Cry1Ac in resistant *P. xylostella* strains was tightly related to the differential expression of two *PxABCC* genes in the *BtR-1* locus and a *PxmALP* gene outside of this locus [26]. Furthermore, the functional redundancy between the ABC transporters (PxABCC2/3) and two additional downregulated GPI-anchored APN (PxAPN1/3a) proteins required all four to be removed to elicit a high (> 34,000 fold) resistance to Bt Cry1Ac toxin [32]. To further investigate the role of GPI-anchored *PxmALP* in the mode of action of Bt toxins, we generated *P. xylostella* single and triple KO strains (ALPKO and ALP-C2C3KO) by introducing a *PxmALP* knockout in the susceptible DBM1Ac-S and previously created C2-3KO strains, respectively (Fig. 1a).

**Fig. 2:**
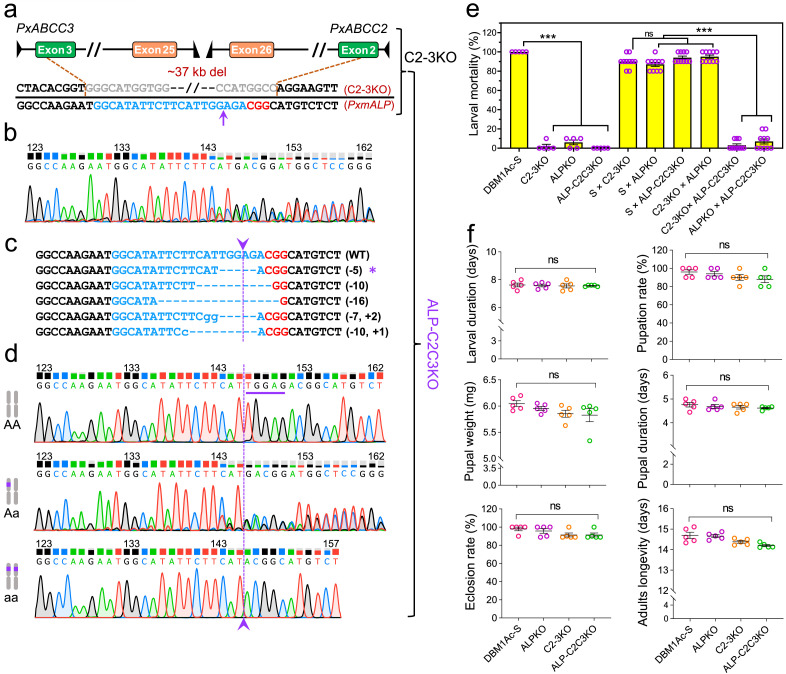
**CRISPR/Cas9-mediated triplex gene mutation of *PxABCC2*/*PxABCC3*/*PxmALP*.** (a) Construction of three-gene mutant strains using C2-3KO as the parental strain. The sequence structure of the previously established C2-3KO strain. (b) CRISPR/Cas9-based gene mutations in G0 individuals were confirmed by direct sequencing. (c) The exact mutation genotypes in G1 individuals were further verified by TA cloning and sequencing. Among these different mutation genotypes, the deleted bases are indicated as dashes, and the inserted bases as lowercase letters. The number of deleted and inserted bases is shown at the right of each allele. Asterisk represents the selected monoallelic mutation used for crossing to yield G2 progeny. (d) Representative chromatograms of direct sequencing of the PCR products from wild types, heterozygous, and homozygous mutants of the *PxmALP* gene. The CRISPR/Cas9-induced 5-bp deletion (TGGAG) in exon 2 of the *PxmALP* gene is labeled by a purple line and arrow (shown from top to below). (e) Interstrain allelic complementation analysis with a diagnostic dose of Cry1Ac protoxin. F1 progenies were generated by six interstrain crosses between DBM1Ac-S, C2-3KO, ALPKO, and ALP-C2C3KO. The mortality of the parental strains (50 larvae from each strain) and their F1 offspring (100 larvae from each F1 hybrid progeny) was tested with a diagnostic dose (10 mg/L) of Cry1Ac protoxin, which was previously determined to kill 100% of susceptible and heterozygotes larvae. (f) Fitness parameters in multiple genome editing strains. A series of biological parameters were compared in resistant KO strains (C2-3KO, ALPKO, and ALP-C2C3KO) with the susceptible DBM1Ac-S strain as control, including larval duration time, pupation rate, pupal weight, pupal duration time, eclosion percentage, and adults’ longevity. Significant differences in b and c were analyzed by one-way ANOVA. The *p* values from Holm-Sidak’s test, *p < 0.05, **p < 0.01, ***p < 0.001, ns, no significant. Data are presented as mean value ± SEM for C, *n* = 5 biologically independent samples with ten larvae per replicate.

A total of 203 and 192 fresh pre-blastoderm eggs from the DBM1Ac-S and C2-3KO strains were respectively collected and injected with a mixture of Cas9 protein and sgRNA targeting exon 2 of *PxmALP* (Figs. 1c, 2a). After injection, approximately 67% (135/203) of the injected eggs hatched, and 62% (84/135) of them developed into adults for the *PxmALP*-KO group (group 1) during G0 (Table 1). In the *PxABCC2/3/mALP*-KO group (group 2), 63% (121/192) of the injected eggs hatched, and 58% (70/121) of them successfully developed into adults during G0. Subsequently, the nondestructive genotyping and direct sequencing assay described in Materials and Methods was used to screen for potential mutations. Analysis of the chromatograms of direct sequencing indicated that 68% (57/84) and 70% (49/70) of the detected G0 individuals in groups 1 and 2 respectively contained mutations. The mutant moths in each group were sib-crossed with each other to generate G1 progeny (Figs. 1d, 2b; Table 1).

**Table 1:** **CRISPR/Cas9-induced mutations in DBM1Ac-S and C2-3KO**.

Original strains	G0				G1			G2	Mutant strain
	Eggs#	Hatched (%)	Adults (%)	Mutant (%)*	Pupae	Genotypes (%)‡	Sib-cross†	Homozygous (%)¶	
DBM1Ac-S	203	135/203 (67)	84/135 (62)	57/84 (68)	100	57/100 (57)	—	—	ALPKO
						17/100 (17)	—	—	
						35/100 (35)	−4 (19)**√**	27 (12♂, 15♀)	
							−5 (8)	—	
							−13 (6)	—	
							−4, +3 (1)	—	
							−8, +3 (1)	—	
C2–3KO	192	121/192 (63)	70/121 (58)	49/70 (70)	108	55/108 (51)	—	—	ALP-C2C3KO
						19/108 (18)	—	—	
						34/108 (31)	−5 (22)**√**	18 (10♂, 8♀)	
							−10 (6)	—	
							−16 (3)	—	
							−7, +2 (2)	—	
							−10, +1 (1)	—	

# A total of 203 and 192 fresh preblastoderm eggs from the DBM1Ac-S and C2–3KO strains were collected for microinjection.

⁎ Nondestructive genotyping of indel mutations surrounding the sgRNA target site was performed by PCR, 68% (57/84) and 70% (49/70) individuals showed site-specific mutagenesis efficiency in G0 offspring.

‡ A total of 100 and 108 pupae of G1 were obtained for genotyping, three genotypes were included in both groups. For the ALPKO group, 57% (57/108), WT; 17% (17/108), biallelic heterozygous mutants; 35% (35/100), monoallelic mutants. For the ALP-C2C3KO group, 51% (55/108), WT; 18% (19/108), biallelic heterozygous mutants; 31% (34/108), monoallelic mutants.

† Among ALPKO and ALP-C2C3KO groups, the 35/34 monoallelic mutants also included 5 different mutation types, the largest number of individuals were mass-crossed with each other to generate G2 progeny.

¶ 27 (15 males and 12 females) and 18 (8 males and 10 females) homozygous mutants were mated to generate ALPKO and ALP-C2C3KO strains.

About 100 4th-instar larvae of G1 from groups 1 and 2 (sex ratio = 1:1) were selected for mutation detection by extracting gDNA from the exuviates of these larvae. Direct sequencing was performed to identify the genotypes of G1 individuals and TA-clone sequencing was conducted to confirm the exact sequence of these mutations (Figs. 1e, 2c). Subsequently, from the groups containing the largest numbers of insects with the same mutation (9 males and 10 females with a 4-bp deletion in group 1, 10 males and 12 females with a 5-bp deletion in group 2) adults were mass crossed with each other to produce G2 offspring (Figs. 1f, 2d). Sequencing of randomly screened exuviates from G2 pupae revealed that 24% (27/112 in group 1) and 18% (18/98 in group 2) were homozygous for the respective deletions and both mutations were predicted to introduce stop codons that resulted in non-functional proteins. Ultimately, 27 (15 males and 12 females) and 18 (8 males and 10 females) homozygous mutants from groups 1 and 2 respectively were sib-mated to generate two stable homozygous mutant strains designated ALPKO and ALP-C2C3KO (Fig. 1b; Table 1).

### 3.2 Resistance to Bt Cry1A protoxins caused by gene KO

Previously, we had shown that knocking out *PxABCC2* or *PxABCC3* individually only had a small effect on the susceptibility of *P. xylostella* to Cry1Ac, whereas a significantly larger effect was seen when both proteins were removed [25]. Also, we had shown that the simultaneous mutation of both ABC transporters and two GPI-anchored APN proteins resulted in extremely high levels of resistance to Cry1Ac toxin [32]. To determine the potential interaction of PxmALP with the ABC transporters, bioassays were conducted to assess the susceptibility to Cry1A toxin in the ALPKO and ALP-C2C3KO strains, compared to the susceptible DBM1Ac-S strain (Table 2).

**Table 2:** **Resistance to Cry1A toxins in larvae from DBM1Ac-S, C2–3KO, ALPKO, and ALP-C2C3KO**.

Toxins	Strain	N*	LC_50_ (95% CL)†	Slope ± SE	χ^2^(df)‡	RR§
Cry1Ac	DBM1Ac-S	210	0.73 (0.56–0.93)	2.02 ± 0.24	3.14(5)	1.00
	C2-3KO	210	3,281.11 (2,612.45–4,208.60)	2.34 ± 0.28	3.74(5)	4,494.67
	ALPKO	210	214.35 (136.97–353.04)	2.38 ± 0.27	10.7(5)	293.63
	ALP-C2C3KO	210	7,051.58 (5,420.84–10,050.46)	2.20 ± 0.34	1.22(5)	9,659.69
Cry1Ab	DBM1Ac-S	210	0.78 (0.59–1.01)	1.85 ± 0.22	4.10(5)	1
	C2-3KO	210	440.18 (335.35–610.77)	1.86 ± 0.24	0.55(5)	564.33
	ALPKO	210	307.70 (233.20–422.01)	1.72 ± 0.21	1.73(5)	394.49
	ALP-C2C3KO	210	4,415.97 (3,346.89–6,184.52)	1.82 ± 0.24	0.73(5)	5,661.5
Cry1Aa	DBM1Ac-S	210	0.70 (0.54–0.91)	1.94 ± 0.23	1.90(5)	1
	C2-3KO	210	0.86 (0.66–1.10)	1.97 ± 0.23	2.50(5)	1.23
	ALPKO	210	0.83 (0.62–1.10)	1.67 ± 0.20	3.70(5)	1.20
	ALP-C2C3KO	210	0.80 (0.62–1.03)	1.99 ± 0.23	1.72(5)	1.14

⁎ Number of larvae tested (larvae of the control group not included).

† Concentration of Cry1Ac toxin (mg/L) killing 50% of larvae and its 95% confidence limits (CL).

‡ The value of chi-square and degrees of freedom (df) were calculated by Polo Plus 2.0.

§ RR: Resistance ratio (RR) calculated by LC_50_ of resistant divided by LC_50_ of DBM1Ac-S.

The results showed that the resistance ratios of ALPKO, C2-3KO, and ALP-C2C3KO strains to Cry1Ac protoxin were 294-, 4,495-, and 9,660-fold respectively (Fig. 5; Table 2). These findings are consistent with previous studies [32], and indicate that while individual receptor knockouts result in some decrease in susceptibility, multiple knockouts are required to produce significant decreases. The resistant KO strains also exhibited notable cross-resistance to Cry1Ab protoxin (ALPKO: 394-fold, C2-3KO: 564-fold, ALP-C2C3KO: 5,662-fold) but did not show cross-resistance to Cry1Aa protoxin (ALPKO: 1.20-fold, C2–3KO: 1.23-fold, ALP-C2C3KO: 1.14-fold).

### 3.3 Expression of recombinant PxmALP protein in insect Sf9 cells

**Fig. 3:**
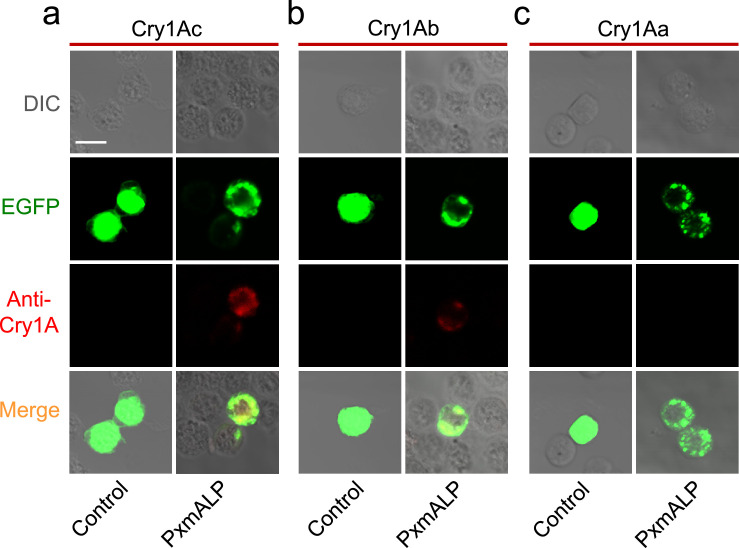
**PxmALP is a receptor for Bt Cry1Ac and Cry1Ab but not for Cry1Aa.** (a) Immunofluorescence detection of Cry1Ac binding to Sf9 cells expressing the PxmALP-EGFP fusion protein. (b) Detection of Cry1Ab binding to Sf9 cells expressing PxmALP-EGFP. (c) Detection of Cry1Aa binding to Sf9 cells expressing PxmALP-EGFP. Gray panels: differential interference contrast (DIC) microscopy views; green panels: fluorescence of EGFP-fusion proteins; red panels: Cry1A-binding fluorescent signal with rabbit polyclonal anti-Cry1A primary antibody and goat anti-rabbit secondary antibody conjugated with red fluorescence Alexa Fluor 555; superimposed panels: merged images from both the green and red fluorescent channels. The scale bar is 20 µm.

**Fig. 4:**
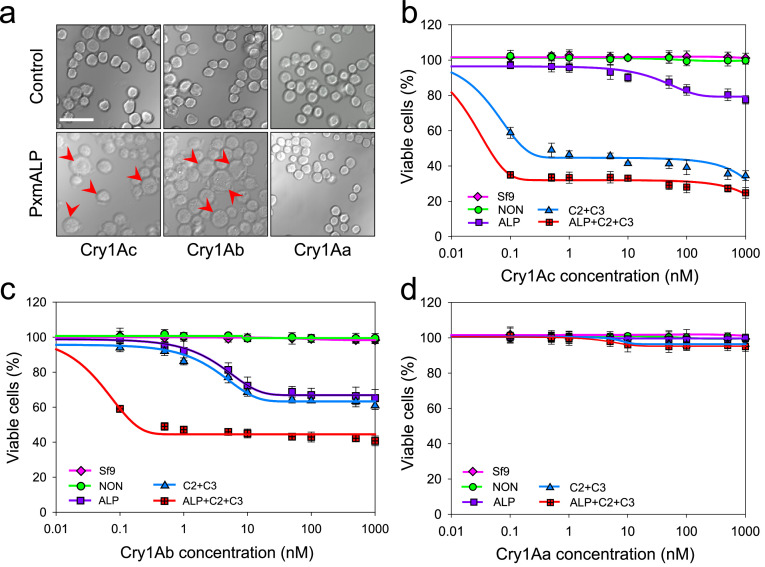
**The cytotoxicity assay in Sf9 cells expressing PxABCC2-3/PxmALP recombinant proteins treated with Cry1A toxin.** (a) Morphological changes of Sf9 cells after exposure to Cry1Ac toxin (50 nM). Cells were observed and representative regions at 24 h after incubation with Cry1Ac toxin are shown. (b–d) Susceptibility to different concentrations of Cry1A toxin (0.1 nM-1 µM) in untransfected Sf9 cells (Control), Sf9 cells transfected with an empty expression-vector (NON), PxmALP-expressing Sf9 cell, and Sf9 cell with simultaneous expression of multiple proteins (both PxABCC2 and PxACCC3, and PxmALP, PxABCC2 and PxACCC3). Cell viability was detected by CCK-8 assay after 24 h incubation with different concentrations of Cry1A toxin. The data are indicated as mean ± SEM. The red arrows point to the damaged cells. The scale bar is 20 µm.

To further analyze the role of *PxABCC2-3* and *PxmALP* in Cry1Ac toxicity, these genes were transiently expressed in insect Sf9 cells by transfection of the relevant recombinant plasmids (pie2-EGFP-*PxABCC2*, pie2-EGFP-*PxABCC3*, and pie2-EGFP-*PxmALP*). The localization of EGFP-tagged *PxmALP* in transfected Sf9 cells was examined using laser confocal microscopy. The results showed that Sf9 cells expressing only EGFP exhibited localization throughout the cell, while the expression of PxmALP-EGFP was located primarily on the cell surface and partially in the cytoplasm (Fig. 3). Subsequently, Sf9 cells expressing the recombinant proteins were incubated with trypsin-activated Cry1Ac, Cry1Ab and Cry1Aa toxins, and the location of these toxins was revealed with anti-Cry antibodies. This showed that Cry1Ac and Cry1Ab, but not Cry1Aa, could be observed on the cell surface of PxmALP-expressing cells (Fig. 3a, 3b), indicating that PxmALP can serve as a receptor for Cry1Ac and Cry1Ab but not for Cry1Aa (Fig. 3c). Sf9 cells expressing *PxmALP* also showed swollen irregular-shaped soma and lysis after treatment with Cry1Ac and Cry1Ab toxins, but not with Cry1Aa (Fig. 4a).

To confirm that the expressed proteins were acting as functional receptors for the Cry1A toxins, cytotoxicity assays were performed on transfected cells. Cells expressing only EGFP (control) or without transfection (Non) were tolerant to activated forms of all the Cry1A toxins (Fig. 4a–d). The PxmALP-expressing cells showed a small, but significant, increase in susceptibility to both Cry1Ab and Cry1Ac (but not Cry1Aa) (Fig. 4b–d), which mirrored the small increase in resistance ratio to the former two toxins in the ALPKO strain (Table 2). The response of the PxABCC2/3-expressing cells also reflected the knockout data with a larger increase in susceptibility/resistance ratio being seen for Cry1Ac than for Cry1Ab. As before, no effect was seen with Cry1Aa. With cells expressing all three receptor genes a strong correlation with resistance ratio was again seen with the response to Cry1Ab and Cry1Ac being broadly similar but greater than that seen in cells expressing just PxmALP or PxABCC2/3. These results confirm that PxmALP can act as a functional receptor for Cry1Ab and Cry1Ac and can work in tandem with PxABCC2 and PxABCC3.

### 3.4 Genetic inheritance of resistance to Cry1Ac in the KO strains

The mode of inheritance of Cry1Ac resistance in the KO strains was investigated by crossing DBM1Ac-S, ALPKO, C2-3KO, and ALP-C2C3KO pairs to obtain F1 progeny. Larvae were then exposed to a diagnostic dose of Cry1Ac protoxin (10 mg/L), which would kill all susceptible and heterozygous moths. The results indicated that the mortality was 100% in the susceptible DBM1Ac-S strain while all the resistant KO strains had a high survival rate (92%–100%) (Fig. 2e). Also, the F1 progeny produced by hybridization of DBM1Ac-S and any of resistance KO strain had a lower survival rate (6%–10%), similar to the F1 progeny from crosses between ALPKO and C2-3KO strains (5%). On the contrary, F1 progeny from crosses of the triple (ALP-C2C3KO) and double (C2-3KO) mutants or from the triple with the single (ALPKO) mutant strains exhibited high survival rates (93%–97%) (Table S2). These results confirm that ALP-C2C3KO shares some of the same resistance alleles with C2-3KO and ALPKO.

### 3.5 Fitness costs caused by genome editing

Our previous results found that the quadruple KO strain C-NKO (*PxABCC2*/*PxABCC3*/*PxAPN1*/*PxAPN3a* KO) showed fitness costs for a series of biological parameters, which were not observed in the two double C2-3KO (*PxABCC2*/*PxABCC3* KO) and N1-3aKO (*PxAPN1*/*PxAPN3a* KO) knockout strains [32]. To further identify whether the individuals from the single (ALPKO) or triple (ALP-C2C3KO) mutant strains had fitness disadvantages, multiple biological parameters were evaluated (Fig. 2f). Our analysis revealed no fitness costs in the ALPKO or ALP-C2C3KO mutants.

## 4 Discussion and conclusion

Research in recent years has clearly established that in a given insect multiple functional receptors can exist for a particular Cry toxin [40]. This naturally leads to questions concerning the relative role of individual receptors both in the mechanism of action of the toxin and in the development of resistance. Do multiple receptors co-operate in the mechanism of action? How many receptors have to be inactive in order to get functional resistance? The creation of CRISPR/Cas9 gene knockouts alongside ectopic expression of receptors in otherwise non-susceptible cells are powerful tools that can be used to address these questions. The data presented in this study, and previously, have demonstrated that particular receptor proteins from *P. xylostella* can individually function as receptors for a Cry protein but also that the combined effect of two or more is often greater than the additive effect of the individual components. For example, in this work, we show that knocking out *PxABCC2*/*3* or *PxmALP* gives resistance ratios of 564 and 394 respectively to Cry1Ab (Table 2) whereas knocking them out altogether gives a ratio of 5,661. Similarly, expressing the proteins separately in Sf9 cells does not result in significant loss of viability when treated with 0.1 nM Cry1Ab (Fig. 4c) whereas expressing all does. This represents the classical definition of synergism but does not provide a mechanistic explanation. There is not a linear relationship between receptor concentration and susceptibility and not knowing this relationship makes it difficult to properly interpret the data, for instance, it is easy to imagine that for a well-expressed receptor removing half of the molecules may not have a significant effect on susceptibility whereas removing all of them would clearly have a major effect. Thus, the synergistic effects described above do not imply that there is an interaction between two receptor types, but neither do they rule them out. Potential interactions that could take place between receptors have been discussed in detail in several recent reviews [15,40].

**Fig. 5:**
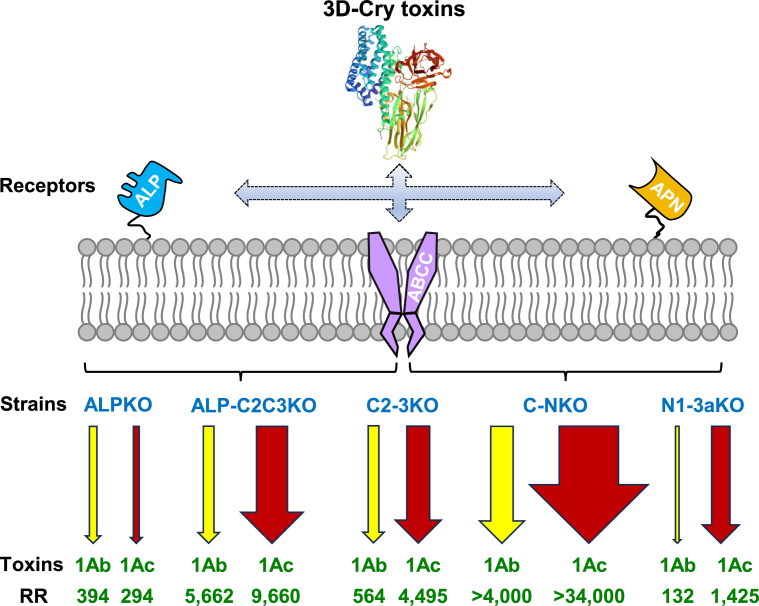
**Interaction of Cry1Ab and Cry1Ac with *P. xylostella* midgut receptors.** Diagram summarizing the effect of knocking out groups of receptors on resistance levels to Cry1Ab and Cry1Ac. ALP refers to PxmALP, ABCC to PxABCC2+3 and APN to PxAPN1+3a, the thickness of the arrows indicates the relative resistance ratios whose values are shown below each arrow. The figure indicates some combinations of receptors that have been knocked out. Different *P. xylostella* strains: ALPKO, ALP-C2C3KO, C2-3KO, C-NKO, and N1-3aKO indicate CRISPR/Cas9-mediated knockout of *PxmALP, PxmALP*/*PxABCC2*/*PxABCC3, PxABCC2*/*PxABCC3, PxABCC2*/*PxABCC3*/*PxAPN1*/*PxAPN3a*, and *PxAPN1*/*PxAPN3a* genes*,* respectively. RR: Resistance ratio.

The data presented in Table 2 and Fig. 1 show significant differences in the response of the knockouts and transgenic cell lines to the three different Cry1A toxins. Such differences are consistent with the individual toxins binding to different receptors or combinations of receptors. The use of competitive binding studies predicted the presence of multiple receptors and early models suggested that there were at least two receptors for Cry1Aa, Cry1Ab, and Cry1Ac with all three being able to bind to a common receptor while Cry1Aa could also bind to an alternative one [41]. Such a model is consistent with the data obtained in this study with Cry1Aa. If the unique Cry1Aa receptor is not ABCC2, ABCC3, or ALP then that would explain why the knockouts did not reduce susceptibility or the transgenic cells acquire susceptibility. Although the competitive binding data suggested a common receptor for the three Cry1A toxins, if this was an inefficient receptor for Cry1Aa, then knocking it out would have little effect on this toxin. We had previously also shown that knockouts of *APN1* and *APN3a* had no effect on Cry1Aa susceptibility [32]. A revised model for Cry toxin binding to *P. xylostella* receptors [42] suggested that as well as binding to the communal receptor Cry1Ab and Cry1Ac could also bind to a third receptor to which Cry1Aa could not. Our data indicate that there are differences between the responses to Cry1Ab and Cry1Ac in particular with respect to ABCC2/3. Knocking out these two receptors increases the resistance ratio to Cry1Ac to 4,495 but only to 564 for Cry1Ab, similarly Sf9 cells expressing ABCC2/3 are much more sensitive to Cry1Ac than to Cry1Ab. These indicate that the ABCC proteins are more effective receptors for Cry1Ac than for Cry1Ab. In the resistant NIL-R strain [46], in which multiple receptors are downregulated, high resistance ratios to both Cry1Ac and Cry1Ab are seen (Table S3), but given the many factors that could influence susceptibility in this strain, no meaningful conclusions can be drawn from the ratios regarding the relative contribution of different receptors. Fig. 5 summarizes these data and graphically represents the relative contribution of the knockouts to the resistance phenotype for these two toxins. The effectiveness of a receptor towards a given toxin could depend on a number of factors such as strength of binding [40] or orientation of binding [43].

*P. xylostella* was the first insect to develop resistance to Bt products in the field and a pattern of resistance in which the insect was resistant to Cry1A toxins, showed reduced binding of these toxins, but had no cross-resistance to Cry1Ca was defined as “Mode 1” [44]. Initially characterized strains showed resistance to Cry1Aa, Cry1Ab and Cry1Ac with resistance thought to be due to a single locus [45]. To date, we have not identified a single receptor mutation that would give resistance to these three toxins, though one might exist. One has though been identified that results in downregulation of multiple receptors [46,47] and shows resistance to Cry1Aa (Table S3), the indications are that the initial resistant strains were as a result of a mutation in ABCC2 [48], and a derived strain has subsequently been shown to be resistant without showing the downregulation phenotype [49]. The lack of correlation between the presence of PxABCC2 and susceptibility to the three Cry1A toxins just shows that while we now have a much better understanding of the relationship between insect susceptibility and expression of particular receptors the overall picture remains as unclear as it ever was [50,51].

## CRediT authorship contribution statement

DS, NC and ZG designed the research; DS, QX, LG, YB, NC and ZG performed the research; DS, NC and ZG analyzed the data; DS, XS, XY, NC, XZ, AB, MS, YZ and ZG wrote and revised the paper.

## Declaration of competing interest

The authors declare that they have no conflicts of interest in this work.

## Appendix Supplementary materials

Image, application 1
