# Supplementary material for: The role of GPI-anchored membrane-bound alkaline phosphatase in the mode of action of Bt Cry1A toxins in the diamondback moth
Source: Fundam Res. 2024 May 27;5(2):674–82. doi: 10.1016/j.fmre.2024.05.007 (PMC11997606; doi:10.1016/j.fmre.2024.05.007)
Supplement: Supplementary file 1 [file mmc1.docx]

**Supplementary Materials**

**The role of GPI-anchored membrane-bound alkaline phosphatase in the mode of action of Bt Cry1A toxins in diamondback moth**

Dan Sun^a,1^, Qiuchen Xu^a,1^, Le Guo^b^, Yang Bai^b^, Xuping Shentu^a^, Xiaoping Yu^a^, Neil Crickmore^c^, Xuguo Zhou^d^, Alejandra Bravo^e^, Mario Soberón^e^, Youjun Zhang^b^, Zhaojiang Guo^b,^*

^a^ Zhejiang Provincial Key Laboratory of Biometrology and Inspection & Quarantine, College of Life Science, China Jiliang University, Hangzhou 310018, China.

^b^ State Key Laboratory of Vegetable Biobreeding, Department of Plant Protection, Institute of Vegetables and Flowers, Chinese Academy of Agricultural Sciences, Beijing 100081, China.

^c^ School of Life Sciences, University of Sussex, Brighton BN1 9QG, UK.

^d^ Department of Entomology, School of Integrative Biology, College of Liberal Arts & Sciences, University of Illinois Urbana-Champaign, Illinois 61801-3795, USA.

^e^ Departamento de Microbiología Molecular, Instituto de Biotecnología, Universidad Nacional Autónoma de México, Apdo. Postal 510-3, Cuernavaca, 62250, Morelos, México.

^1^These authors contributed equally to this work.

*Corresponding author: guozhaojiang@caas.cn (Z. Guo).

**Table S1.** Primers used in this study

| Purpose | Gene name | Primer name | Primer sequence (5´-3´) | PCR product (bp) | Tm (°C) |
| --- | --- | --- | --- | --- | --- |
| CRISPR sgRNA* | PxmALP | CRISPR-ALP-F | *GAAATTAATACGACTCACTATAGG****GGCATATTCTTCATTGGAGA****GTTTTAGAGCTAGAAATAGC* | 124 | 70 |
|  |  | CRISPR-R | AAAAGCACCGACTCGGTGCCACTTTTTCAAGTTGATAACGGACTAGCCTTATTTTAACTTGCTATTTCTAGCTCTAAAAC |  |  |
| Specific sequencing primers^§^  Full-length cDNA cloning | PxmALP | ALP-F | AAGATGTGGCCACACCGAAACC | 291 | 57 |
|  |  | ALP-R | GCCCGCTGACAGGAAACTTCTC |  |  |
|  | PxABCC2 | C2-F-F | ATGGAAAACGGAAGCGGAGC | 4041 | 63 |
|  |  | C2-F-R | TTGAGGATGGTCGTCGAAGTATTTC |  |  |
|  | PxABCC3 | C3-F-F | ATGGGGGTGAAGGTTGCGGA | 4044 | 62 |
|  |  | C3-F-R | CCTAGCGTTCTCTTTCATATTGCTT |  |  |
|  | PxmALP | ALP-F-F | ATGTCTCGCGTGGCGCGCCA | 1683 | 60 |
|  |  | ALP-F-R | TAATAAGCGTCTCAGATACGAAACA |  |  |
|  | PxABCC1 | C1-EXP-F | *CCC*AAGCCTATGGTGAGTGGTAATAAGGACCCGT | 4182 | 63 |
| Heterologous expression^‡^ | PxABCC2 | C2-EXP-F | *CCC*AAGCCTATGGAAAACGGAAGCGGAGC | 4059 | 63 |
|  |  | C2-EXP-R | *CTA*GCTAGCTTGAGGATGGTCGTCGAAGTATTTC |  |  |
|  | PxABCC3 | C3-EXP-F | *CCC*AAGCCTATGGGGGTGAAGGTTGCGGA | 4062 | 62 |
|  |  | C3-EXP-R | *CTA*GCTAGCCCTAGCGTTCTCTTTCATATTGCTT |  |  |
|  | PxmALP | ALP-EXP-F | *CCC*GCTAGCATGTCTCGCGTGGCGCGCCA | 1701 | 62 |
|  |  | ALP-EXP-R | *CTA*AAGCTTTAATAAGCGTCTCAGATACGAAACA |  |  |

*A specific oligonucleotide encoding a T7 polymerase-binding site (italicized) and the sgRNA target sequence (underlined and bold) of *PxmALP* was designed as the forward primer CRISPR-F, and a common oligonucleotide encoding the remaining sequences of CRISPR-F were designed as the reverse primer CRISPR-R.

^§^To further verify the accurate information of the indel sequences, we amplified the genome DNA fragment (291 bp) of *PxmALP* gene flanking CRISPR target site by using the exuviate gDNA of 4^th^-instar *P. xylostella* larvae as a template with the specific sequencing primer, and the PCR products of the mutants were ligated into *pEASY*-T1 cloning vector and sequenced for validation of the genomic mutated events.

^‡^Using restriction enzymes *Hind*III and *Nhe*I to cut the pie2-GFP-N1 expression vector and generate a linearized vector, then the protective bases CCC (italicized) and restriction endonuclease *Nhe*I (underlined) were added before the upstream primers of PxABCC2-3-Exp-F and PxmALP-Exp-F, similarly, the protective bases CTA (italicized) and restriction endonuclease *Hind*III (underlined) were added after the downstream primers of PxABCC2-3-Exp-R and PxmALP-Exp-R. Genes cloned into the multiple cloning site (MCS) will be expressed as PxABCC2-3-GFP or PxmALP-GFP fusion proteins.

**Table S2**. Toxicity to Cry1Ac toxin in larvae from the various knockout strains and their F1 progeny

| Strains* | Survival rate (%)^†^ | Dominance (*h*)^§^ |
| --- | --- | --- |
| DBM1Ac-S | 0 |  |
| C2-3KO | 98 |  |
| ALPKO | 92 |  |
| ALP-C2C3KO | 100 |  |
| DBM1Ac-S × C2-3KO^#^ | 10 | 0.10 |
| DBM1Ac-S × ALPKO^#^ | 8 | 0.09 |
| DBM1Ac-S × ALP-C2C3KO^#^ | 6 | 0.06 |
| C2-3KO × ALPKO^‡^ | 5 |  |
| C2-3KO × ALP-C2C3KO^‡^ | 97 |  |
| ALPKO × ALP-C2C3KO^‡^ | 93 |  |

*Reciprocal crossing (50 males and 50 females) was performed among DBM1Ac-S, C2-3KO, ALPKO, and ALP-C2C3KO strains.

^†^The sample size to obtain the survival rate was 50 larvae for each parent strain and 100 larvae for their F1 progeny.

^§^The dominance parameter *h* was calculated by the survival rate at 10 mg/L of Cry1Ac toxin as (the survival rate of F1 hybrid progeny – the survival rate of DBM1Ac-S) divided by (the survival rate of resistant strain – the survival rate of DBM1Ac-S). As mentioned elsewhere, the *h* value varies from 0 (completely recessive resistance) to 1 (completely dominant resistance).

^#^Toxicity to Cry1Ac toxin in F1 larvae produced by crossing the KO strain and the susceptible DBM1Ac-S strain.

^‡^Toxicity to Cry1Ac toxin in F1 larvae produced by crossing the different KO strains.

**Table S3**. Resistance to Cry1A toxins in larvae from the DBM1Ac-S and NIL-R strains.

| Toxins | Strains | N* | LC_50_ (95% CL)^†^ | Slope ± SE | χ^2^(df)^‡^ | RR^§^ |
| --- | --- | --- | --- | --- | --- | --- |
| Cry1Ac | DBM1Ac-S | 210 | 0.76 (0.60-0.97) | 2.17 ± 0.25 | 2.30(5) | 1.0 |
|  | NIL-R | 210 | 3898.55 (2627.09-6432.54) | 2.21 ± 0.28 | 7.77(5) | 5129.67 |
| Cry1Ab | DBM1Ac-S | 210 | 0.71 (0.54-0.92) | 1.84 ± 0.22 | 1.24(5) | 1.0 |
|  | NIL-R | 210 | 1917.09 (1473.56-2529.24) | 1.81 ± 0.21 | 4.94(5) | 2700.27 |
| Cry1Aa | DBM1Ac-S | 210 | 0.70 (0.55-0.88) | 2.26 ± 0.27 | 3.96(5) | 1.0 |
|  | NIL-R | 210 | 108.30 (84.87-139.99) | 2.04 ± 0.24 | 4.84(5) | 154.71 |

*Number of larvae tested (larvae of the control group not included).

^†^Concentration of Bt Cry1A toxins (mg/L) killing 50% of larvae and its 95% confidence limits (CL).

^‡^The value of chi-square and degrees of freedom (df) were calculated by Polo Plus 2.0.

^§^RR: Resistance ratio (RR) calculated by LC_50_ of resistant divided by LC_50_ of DBM1Ac-S.
